# Analysis on genetic diversification and heterosis in autotetraploid rice

**DOI:** 10.1186/2193-1801-2-439

**Published:** 2013-09-05

**Authors:** Jin-Wen Wu, Chao-Yue Hu, Muhammad Qasim Shahid, Hai-Bin Guo, Yu-Xiang Zeng, Xiang-Dong Liu, Yong-Gen Lu

**Affiliations:** State Key Laboratory for Conservation and Utilization of Subtropical Agro-bioresources, South China Agricultural University, Guangzhou, 510642 China

**Keywords:** Genetic distance, Genetic variation, Heterobeltiosis, Inter-subspecific hybrids, Polyploidy

## Abstract

Polyploidization has played an important role in plant evolution and is a pathway for plants to increase genetic diversification and to get higher heterosis comparing with that of diploid does. This study was undertaken to assess the genetic variation and relationships among 40 autotetraploid rice genotypes and their counterpart diploid cultivars with 99 SSR markers screened from published rice genome. The 99 SSR markers detected polymorphism among autotetraploid genotypes and revealed a total of 291 alleles with an average of 2.949 alleles per locus. Autotetraploid lines showed higher genetic diversity and significant variation in agronomic traits than diploid cultivars. Phylogenetic analysis revealed that most of autotetraploid lines were genetically different from their diploid parents, and inter-subspecific hybrids were prepared on the basis of genetic distance between parents. Inter-subspecific autotetraploid hybrids showed a higher and positive heterobeltiosis and competitive heterosis than diploid hybrids, especially for grain yield. Genetic distance appeared not to predict heterosis in diploid rice for all traits; however, it showed a significant correlation with grain yield, grain length and grain length to width ratio in autotetraploid rice. This extensive research on autotetraploid heterosis and genetic diversity will be useful for the development of autotetraploid rice hybrids.

## Introduction

Polyploidization has played an important role in plant evolution and is a pathway for plants to increase genetic diversification and to get higher heterosis comparing with that of diploid does (Doyle et al. 2008;Luan et al. 2008;Shahid et al. 2012). Over 70% of all angiosperm species have an increase in ploidy level somewhere during their evolutionary histories (Masterson 1994). Polyploid species with doubling genomes showed abundant advantages for various traits, such as greater variation, high biomass yield and resistance to insect pest and diseases (Bingham et al. 1994;Marhold and Lihová^2006^).

Autotetraploid rice is a new germplasm developed from diploid rice through chromosome doubling with colchicine treatment and has the potential to increase rice production and nutrition (Song and Zhang 1992;Shahid et al. 2012). Autotetraploid lines showed significantly lower fertility than diploid cultivars (Shahid et al. 2010), but autotetraploid hybrids produced significantly higher fertility than their diploid counterparts (Hu et al. 2009;Shahid et al. 2011). Autotetraploid rice showed higher genetic variation in various agronomic traits than their original diploid rice, such as varying seed set along with longer grains and awns (Li and Rutger 2007;Luan et al. 2008). Inter-subspecific (*indica* × *japonica*) autotetraploid rice hybrids showed more hybrid vigour and stability than diploid hybrids, although a lower seed set is a hindrance in heterosis utilization (Shahid et al. 2011). Autotetraploid rice germplasm for hybrid rice application, including genetic variation and diversity, has not been exploited. Therefore, it is of immense importance to study the genetic variation and to utilize the super heterosis of autotetraploid rice.

Heterosis or hybrid vigor is an important tool for improving the quality and increasing yield of crops since its success in maize. Hybrid breeding has been an important method of increasing grain yield of rice and inter-subspecific crosses, such as *indica* and *japonica*, showed a great potential to raise grain yield than other crosses (Yuan 1987). Most of super rice breeding programs in China have used inter-subspecies heterosis (Cheng et al. 2007). However, the use of heterosis is extremely labour intensive, time consuming, tedious and required a large number of cross-combinations (Zha et al. 2008). The use of molecular markers for prediction of heterosis led into a new phase since its appearance in 1980 (Xiao et al. 1996). A number of studies have been made in various crop plants to predict the relationship between genetic diversity and heterosis, such as diploid rice (Xiao et al. 1995;Zhao et al. 1999;Wang et al. 2013), wheat (Martin et al. 1995), maize (Smith et al. 1990;Betran et al. 2003), barley (Schut et al. 1997) and rape seed (Liu et al. 2002;Yu et al. 2005). Unfortunately, these studies showed two different and contradictory results, some revealed that markers could be used for predicting heterosis, while some other proposed markers could not predict heterosis for complicated traits.

Molecular markers are a powerful tool for the assessment of genetic variability and genetic diversity among genotypes within land races, populations and species. Among PCR-based markers, SSR markers are the most favored in breeding and genetics because of their co-dominant nature, abundance, genome wide coverage and high reproducibility (McCouch et al. 2002). Molecular markers could be used to select parents for hybridization because of the association with different alleles and heterosis (Anand et al. 2012). A number of efforts have been made to investigate the genetic variation of the hybrid parents and heterosis in diploid rice. To the best of our knowledge, there is no report on the relationship of molecular markers genetic distance with hybrid performance in autotetraploid rice. However, there is relatively little known about the detection of genetic variation through SSR markers in autotetraploid rice, which limited the cognition of autotetraploid germplasm in the development of hybrid rice. The objectives of this study were (1) to analyze the genetic variation and genetic distance of autotetraploid and diploid rice using SSR markers, and its relation with heterosis prediction (2) to examine genetic relationship of autotetraploid and corresponding diploid rice cultivars, and (3) to investigate the heterosis of yield and important agronomic traits among autotetraploid and corresponding diploid rice cultivars.

## Materials and methods

### Plant materials

A total of forty autotetraploid rice lines were used to conduct the study about genetic variation, and their 40 diploid parents were used as control (CK) (Table 1). All materials were planted at the experimental farm of South China Agricultural University (SCAU). Row to row (R × R) and plant to plant (P × P) distances were kept as 20 cm and 16.6 cm, respectively. In addition, four *japonica* and seven *indica* autotetraploid rice lines with high genetic diversity were selected based on the results of phylogenetic analysis (Table 2), and then crossed in an incomplete diallel design during 2010. A total of 54 inter-subspecific hybrids (*indica* × *japonica*) of autotetraploid and their counterpart diploid rice were prepared to determine the relationship between hybrid performance and genetic distance. Parents and F_1_ hybrids were planted at the farm of SCAU to conduct the study for heterosis analysis. A Randomized Complete Block Design (RCBD) was used with three replications. R × R and P × P distances were kept as 20 and 16.6 cm, respectively. Seedlings at four-five leaf stage were planted in the paddy field. The F_1_ seeds were harvested from all the crosses at the end of cropping season. All the cultural practices were done according to the recommendations of area.

**Table 1:** **Name of the cultivars with ploidy levels used in this study**

Code	Cultivar	Ploidy	Origion/source	Code	Cultivar	Ploidy	Origion/source
1	Aijiaonante	2×	Guangdong	41	Bo’B	2×	Guangxi
2	Aijiaonante	4×	Lab^a^	42	Bo’B	4×	Lab
3	Guanglu’ai 4	2×	Guangdong	43	Taichung 65	2×	Taiwan
4	Guanglu’ai 4	4×	Lab	44	Taichung 65	4×	Lab
5	L-202	2×	IRRI^b^	45	E2	2×	Guangdong
6	L-202	4×	SCBG- CAS^c^	46	E2	4×	Lab
7	Jackson	2×	IRRI	47	E4	2×	Guangdong
8	Jackson	4×	SCBG- CAS	48	E4	4×	Lab
9	PEDR-2B	2×	Guangdong	49	E5	2×	Guangdong
10	PEDR-2B	4×	Lab	50	E5	4×	Lab
11	Liaojing 944	2×	Liaoning	51	E24	2×	Guangdong
12	Liaojing 944	4×	Lab	52	E24	4×	Lab
13	Yanjing 48	2×	Liaoning	53	E45	2×	Guangdong
14	Yanjing 48	4×	Lab	54	E45	4×	Lab
15	Bengal	2×	IRRI	55	E245	2×	Guangdong
16	Bengal	4×	Lab	56	E245	4×	Lab
17	Raopingsaozhou	2×	Guangdong	57	Lemont	2×	IRRI
18	Raopingsaozhou	4×	Lab	58	Lemont	4×	Lab
19	J455	2×	Guangdong	59	APIV	2×	Lab
20	J455	4×	Lab	60	APIV	4×	Lab
21	Nanhaizaoyinzhan	2×	Guangdong	61	8821	2×	Guangdong
22	Nanhaizaoyinzhan	4×	Lab	62	8821	4×	Lab
23	Yuhei 1	2×	Lab	63	M18	2×	Guangdong
24	Yuhei 1	4×	Lab	64	M18	4×	Lab
25	Xichuan	2×	Guangdong	65	02428	2×	Jiangsu
26	Xichuan	4×	Lab	66	02428	4×	Lab
27	Yuexiangzhan	2×	Guangdong	67	Dalinuo	2×	Guangdong
28	Yuexiangzhan	4×	Lab	68	Dalinuo	4×	Lab
29	Dayebai	2×	Guangdong	69	Huajingxian 74	2×	Guangdong
30	Dayebai	4×	Lab	70	Huajingxian 74	4×	Lab
31	Guinongzhan	2×	Guangdong	71	PII-6	2×	Lab
32	Guinongzhan	4×	Lab	72	PII-6	4×	Lab
33	Shennong 265	2×	Liaoning	73	Shuya	2×	Lab
34	Shennong 265	4×	Lab	74	Shuya	4×	Lab
35	Shennong 15	2×	Liaoning	75	Nanjing 11	2×	Jiangsu
36	Shennong 15	4×	Lab	76	Nanjing 11	4×	Beijing
37	Goulianzao	2×	Guangdong	77	Nantehao	2×	Guangdong
38	Goulianzao	4×	Lab	78	Nantehao	4×	Lab
39	Linglun	2×	Hunan	79	Huayinzhan	2×	Guangdong
40	Linglun	4×	Lab	80	Huayinzhan	4×	Lab

2× indicates diploid rice, 4× indicates autotetraploid rice.

^a^State Key Laboratory for Conservation and Utilization of Subtropical Agro-bioresources, South China Agricultural University, Guangzhou 510642, China.

^b^International Rice Research Institute.

^c^South China Botanical Garden, Chinese Academy of Sciences.

**Table 2:** **Name and types of the parents used to prepare inter-subspecific hybrids**

Sr. #	Diploid parents		Sr. #	Autotetraploid parents	
	Name	Type		Name	Type
1	Liaojing 944–2x	*japonica*	1	Liaojing 944–4x	*japonica*
2	Yanjing 48–2x	*japonica*	2	Yanjing 48–4x	*japonica*
3	Shennong 15–2x	*japonica*	3	Shennong 15–4x	*japonica*
4	Taichung 65–2x	*japonica*	4	Taichung 65–4x	*japonica*
5	Aijiaonante–2x	*indica*	5	Aijiaonante–4x	*indica*
6	Guanglu’ai 4–2x	*indica*	6	Guanglu’ai 4–4x	*Indica*
7	PDER-2B–2x	*indica*	7	PDER-2B–4x	*Indica*
8	Raopingsaozhou–2x	*indica*	8	Raopingsaozhou–4x	*indica*
9	Nanhaizaoyinzhan–2x	*indica*	9	Nanhaizaoyinzhan–4x	*indica*
10	Xichuan–2x	*indica*	10	Xichuan–4x	*indica*
11	Dayebai–2x	*indica*	11	Dayebai–4x	*indica*

### Analysis of agronomic traits

A total of 14 agronomic traits were investigated to find the genetic variation in diploid and autotetraploid rice i.e., plant height (PH, cm), panicle length (PL, cm), effective panicles number (EPN), flag leave length (FLL, cm), flag leave width (FLW, cm), grain length (GL, cm), grain width (GW, cm), grain length to width ratio (L/W), grain density (GD), grain yield (GY), grains per panicle (GPP), total number of grains per plant (TGP), 1000-grain weight (GWT, g) and seed set ratio ((SS = number of filled grains/total number of grains) × 100). Autotetraploid and diploid rice hybrids were planted to examine eleven important agronomic traits: PH, PL, EPN, GL, GW, L/W, GPP, GD, GWT, GY and SS. These traits were selected from the Descriptors and Data Standard for Rice (*Oryza sativa* L.) to describe the genetic variation between autotetraploid and diploid rice cultivars (Han and Wei 2006).

### DNA extraction and SSR analysis

Young leaves were collected from autotetraploid and diploid rice cultivars, and DNA was extracted using modified SDS method (Yang et al. 2009). SSR markers developed by Cornell University and selected from the Gramene database http://www.gramene.org/ were used (Chen et al. 1997;Jaiswal et al. 2006). The volume of the PCR reaction system was 20 μL. The profile of PCR program was as follows: 94°C for 5 min; 30 cycles of 94°C for 1 min, 55°C for 1 min, 72°C for 1 min; and 5 min final extension at 72°C. All amplified products were separated by 6% polyacrylamide gel electrophoresis and detected by silver nitrate staining. Alleles were mainly detected by BIO Imagine System and software Genetools from SynGene and manually re-checked twice.

### Statistical analysis

Agronomic traits data was analyzed using SPSS and the statistical significances were determined using Paired-*T* test by SPSS version 17.0. Levels of heterosis were measured as heterobeltiosis, which is the superiority of a hybrid over the better parent, and competitive heterosis, which is calculated by comparing autotetraploid rice hybrids with their corresponding diploid hybrids. Correlation analysis between the genetic distance and heterobeltiosis was performed with SPSS version 17.0.

Number of effective alleles per locus (*Ae*), expected heterozygosity (*He*) and Shannon’s information index (*I*) were calculated to measure the genetic variation in diploid and autotetraploid rice. Analyses of these computations were assessed using the program POPGENE version 1.32 (Yeh et al. 1997). Polymorphism information content (PIC) was calculated according to the formula: PIC_*i*_ = 1-∑*P*^2^_*ij*_ (*j* = 1) (Anderson et al. 1993) and genetic distance was estimated according to the method of Nei (1978). Neighbor-joining (NJ) tree was constructed by Software NTSYS version 2.10e (Exeter Software, Setauket, NY USA) on the basis of similarity measures (Rohlf 2002), and MEGA V4.0 was used to observe the NJ tree (Tamura et al. 2007).

## Results

### Analysis of agronomic traits

The mean values regarding the agronomic traits of diploid and autotetraploid rice were summarized in Table 3. There were greater differences in the agronomic traits of the diploid and autotetraploid rice. In comparison with the diploid rice, seven traits including GWT, FLL, FLW, PL, GL, GW and L/W showed better performance in autotetraploid than diploid rice. For instance, the mean value of GWT was 24.49 and ranged from 19.60 to 43.03 in diploid rice, while the mean value of GWT was 33.88 in autotetraploid rice and ranged from 28.30 to 52.80. However, EPN, PH, SS, TGP, GPP, GY and GD decreased in autotetraploid rice, such as EPN, the mean value was 7.10 and ranged from 4.33 to 11.00 in diploid rice, while in autotetraploid rice the mean value was 5.09 and ranged from 2.33 to 8.33.

**Table 3:** **Genetic variation in agronomic traits of diploid and autotetraploid rice**

Traits	Diploid			Autotetraploid			t	P
	Mean	Range	CV (%)	Mean	Range	CV (%)		
PH	99.51	66.67–152.00	20.60	90.63	70.83–137.67	15.61	−2.168	0.036*
PL	22.22	14.59–35.87	17.89	25.12	18.58–31.34	10.49	3.684	0.001**
EPN	7.10	4.33–11.00	23.35	5.09	2.33–8.33	31.58	−4.874	1.964E–5**
TGP	514.21	281.00–946.33	29.93	420.17	206.67–704.00	31.17	−3.136	0.003**
GPP	128.07	82.67–229.00	27.10	90.08	54.00–142.56	28.84	−5.279	1.340E–6**
FLL	33.93	18.63–46.90	20.00	37.06	26.33–48.23	14.56	1.763	0.086
FLW	1.68	1.17–2.23	14.19	1.75	1.30–2.27	14.52	1.221	0.230
GL	7.88	6.15–11.97	16.13	9.69	8.00–12.92	11.79	5.677	1.580E–6**
GW	2.84	2.00–3.53	16.40	3.17	2.20–4.10	13.60	4.044	2.478E–4**
L/W	2.89	1.74–5.07	28.98	3.13	2.14–4.83	20.81	1.309	0.198
GD	58.20	37.40–92.90	24.70	35.80	24.4–53.80	25.60	−8.321	4.347E–10**
GY	2.98	0.63–5.74	35.89	1.50	0.11–4.97	72.21	−6.357	1.843 E–7**
GWT	24.49	19.60–43.03	18.26	33.88	28.30–52.80	14.72	9.041	5.209 E–11**
SS (%)	79.29	25.68–96.76	19.36	33.05	2.54–67.57	52.43	−11.282	1.076E–13**

*,** Significantly different from zero at P < 0.05 and P < 0.01, respectively. *CV* = coefficient of variation.

*PH* = plant height, *PL* = panicle length, *EPN* = effective panicles number, *TGP* = total number of grains per plant, *GPP* = grains per panicle, *FLL* = flag leave length, *FLW* = flag leave width, *GL* = grain length, *GW* = grain width, *L/W* = grain length to width, *GD* = grain density, *GY* = grain yield, *GWT* = 1000-grain weight and *SS* = seed set ratio.

In addition, Paired *T*-test was used to evaluate the variation of agronomic traits in different ploidy level. Among all the agronomic traits, we found that PL, TGP, EPN, GWT, GL, GW, GY, SS, GPP and GD showed highly significant (P < 0.01) variation, while PH, showed significant (P < 0.05) variation in ploidy level comparison (Table 3).

### Detection of genetic variation in rice

To detect the genetic variation among the cultivars, 99 microsatellites or simple sequence repeats (SSRs) markers were selected from the rice genome and uniformly distributed on all chromosomes. The results showed a higher variation in autotetraploid rice compared with the diploid rice (Table 4).

**Table 4:** **Genetic diversity and genetic variation detected by SSR markers in diploid and autotetraploid rice**

SSR	Chr^a^	Diploid				Autotetraploid			
Primers		***Ae***^b^	***He***^c^	***I***^d^	PIC^e^	***Ae***	***He***	***I***	PIC
PSM41	1	5	0.735	1.471	0.699	5	0.729	1.451	0.691
RM23	1	3	0.599	0.978	0.513	3	0.647	1.067	0.571
RM443	1	2	0.555	0.882	0.456	3	0.561	0.942	0.493
RM104	1	2	0.469	0.662	0.349	2	0.375	0.562	0.305
RM237	1	2	0.302	0.479	0.256	2	0.332	0.515	0.277
RM262	2	4	0.705	1.303	0.656	4	0.669	1.215	0.613
RM341	2	3	0.599	0.980	0.505	3	0.515	0.824	0.424
RM109	2	3	0.477	0.831	0.428	3	0.453	0.801	0.409
PSM122	2	3	0.619	1.018	0.539	3	0.611	1.011	0.536
RM498	2	3	0.542	0.884	0.457	3	0.492	0.800	0.411
RM29	2	2	0.499	0.693	0.375	2	0.485	0.678	0.368
RM526	2	2	0.498	0.691	0.374	2	0.497	0.690	0.373
RM211	2	2	0.485	0.678	0.368	2	0.455	0.647	0.352
RM106	2	2	0.368	0.555	0.300	2	0.432	0.624	0.339
PSM379	3	5	0.788	1.578	0.753	5	0.681	1.256	0.622
RM168	3	4	0.572	1.062	0.525	4	0.469	0.887	0.431
RM22	3	3	0.559	0.899	0.466	4	0.639	1.162	0.579
RM232	3	3	0.401	0.679	0.345	3	0.384	0.703	0.351
RM156	3	3	0.444	0.780	0.398	3	0.494	0.849	0.438
PSM381	3	3	0.656	1.081	0.581	3	0.662	1.091	0.588
RM565	3	3	0.226	0.461	0.214	3	0.536	0.916	0.478
RM282	3	3	0.609	0.998	0.526	2	0.435	0.627	0.341
PSM429	3	2	0.455	0.647	0.352	2	0.420	0.611	0.332
RM175	3	2	0.049	0.117	0.048	2	0.051	0.122	0.050
RM468	3	2	0.245	0.410	0.215	2	0.307	0.485	0.260
RM416	3	2	0.400	0.589	0.320	2	0.353	0.538	0.291
RM60	3	2	0.051	0.122	0.050	2	0.512	0.613	0.043
RM307	4	5	0.668	1.268	0.608	5	0.498	0.845	0.436
RM241	4	4	0.479	0.879	0.427	4	0.704	1.477	0.647
RM559	4	2	0.129	0.624	0.422	3	0.451	0.722	0.514
PSM194	4	3	0.547	0.932	0.488	3	0.508	0.781	0.404
RM255	4	2	0.293	0.469	0.250	2	0.208	0.362	0.186
RM471	4	2	0.394	0.584	0.317	2	0.495	0.688	0.372
RM261	4	2	0.478	0.671	0.364	2	0.278	0.451	0.239
PSM196	4	2	0.368	0.555	0.300	2	0.496	0.690	0.373
PSM133	4	2	0.260	0.429	0.226	2	0.488	0.681	0.369
RM273	4	2	0.191	0.341	0.173	2	0.412	0.602	0.327
RM164	5	4	0.724	1.327	0.672	4	0.681	1.261	0.632
RM480	5	4	0.698	1.268	0.641	4	0.543	1.010	0.495
RM31	5	4	0.650	1.166	0.585	4	0.719	1.314	0.665
RM122	5	3	0.513	0.785	0.406	3	0.495	0.688	0.372
RM249	5	3	0.579	0.963	0.506	3	0.605	1.008	0.534
PSM383	5	2	0.428	0.619	0.336	3	0.447	0.714	0.368
RM13	5	2	0.497	0.690	0.373	3	0.516	0.785	0.406
RM574	5	2	0.469	0.662	0.359	2	0.478	0.671	0.364
RM527	6	5	0.704	1.346	0.654	5	0.792	1.664	0.787
RM276	6	5	0.747	1.461	0.704	5	0.726	1.433	0.684
RM528	6	3	0.604	1.010	0.536	3	0.639	1.055	0.563
PSM138	6	3	0.566	0.919	0.477	3	0.573	0.922	0.479
RM510	6	3	0.529	0.808	0.418	3	0.570	0.947	0.496
RM340	6	3	0.474	0.807	0.414	4	0.575	1.063	0.526
RM275	6	2	0.149	0.281	0.138	2	0.284	0.458	0.244
RM103	6	2	0.334	0.517	0.278	2	0.368	0.555	0.300
PSM142	7	4	0.725	1.335	0.675	4	0.726	1.339	0.676
RM248	7	4	0.688	1.219	0.624	4	0.645	1.158	0.577
PSM147	7	3	0.528	0.805	0.416	3	0.355	0.540	0.244
RM234	7	2	0.467	0.660	0.358	2	0.415	0.606	0.329
RM560	7	2	0.157	0.293	0.144	2	0.165	0.305	0.496
RM455	7	2	0.450	0.642	0.349	2	0.478	0.671	0.526
RM44	8	4	0.397	0.761	0.363	4	0.260	0.429	0.226
RM210	8	3	0.526	0.836	0.431	3	0.542	0.884	0.684
RM152	8	3	0.539	0.851	0.439	3	0.571	0.918	0.676
RM458	8	2	0.255	0.423	0.223	2	0.420	0.611	0.300
RM408	8	2	0.432	0.624	0.339	2	0.367	0.554	0.329
PSM151	8	2	0.452	0.644	0.350	2	0.426	0.617	0.577
RM126	8	2	0.480	0.673	0.365	2	0.301	0.478	0.255
RM256	8	2	0.049	0.117	0.048	2	0.049	0.117	0.048
RM242	9	4	0.637	1.153	0.573	4	0.477	0.825	0.407
RM257	9	4	0.547	0.997	0.490	3	0.528	0.868	0.449
PSM399	9	3	0.535	0.878	0.454	3	0.374	0.688	0.343
PSM340	9	3	0.595	0.995	0.526	3	0.562	0.917	0.476
RM553	9	3	0.611	1.011	0.536	3	0.447	0.714	0.368
RM434	9	3	0.418	0.719	0.365	3	0.500	0.693	0.375
PSM160	9	2	0.486	0.679	0.368	2	0.482	0.675	0.366
RM591	10	5	0.785	1.572	0.751	5	0.744	1.472	0.704
PSM166	10	3	0.607	0.996	0.525	3	0.586	0.958	0.501
RM258	10	3	0.516	0.860	0.444	2	0.272	0.443	0.235
PSM163	10	2	0.266	0.436	0.231	2	0.420	0.611	0.332
PSM169	10	2	0.500	0.693	0.375	2	0.334	0.517	0.278
RM484	10	2	0.180	0.325	0.164	2	0.139	0.266	0.129
RM202	11	5	0.764	1.518	0.725	5	0.683	1.370	0.647
PSM365	11	5	0.749	1.477	0.708	5	0.727	1.405	0.682
RM224	11	5	0.740	1.467	0.700	5	0.718	1.362	0.666
PSM410	11	4	0.529	0.948	0.465	5	0.704	1.342	0.651
RM229	11	4	0.685	1.228	0.624	4	0.671	1.273	0.604
PSM173	11	3	0.517	0.788	0.408	3	0.532	0.847	0.437
RM167	11	3	0.528	0.805	0.416	3	0.610	1.018	0.541
RM254	11	3	0.538	0.919	0.480	3	0.595	0.995	0.526
PSM411	11	3	0.580	0.943	0.491	3	0.637	1.057	0.565
PSM416	11	2	0.498	0.691	0.374	2	0.500	0.693	0.375
PSM188	12	3	0.586	0.984	0.520	3	0.635	1.046	0.557
RM19	12	3	0.541	0.856	0.432	3	0.553	0.896	0.464
RM101	12	3	0.610	1.001	0.528	3	0.614	1.013	0.536
PSM419	12	3	0.618	1.030	0.549	3	0.514	0.826	0.425
PSM420	12	3	0.141	0.314	0.133	3	0.441	0.706	0.364
PSM187	12	2	0.349	0.533	0.288	2	0.289	0.464	0.247
RM463	12	2	0.293	0.469	0.250	2	0.307	0.485	0.260
PSM191	12	2	0.202	0.355	0.182	3	0.245	0.472	0.226
PSM190	12	2	0.497	0.690	0.374	2	0.498	0.691	0.374
Mean		2.899	0.487	0.819	0.421	2.949	0.493	0.822	0.432
St. Dev		0.953	0.175	0.335	0.162	0.962	0.155	0.319	0.158

^**a**^chromosome number.

^**b**^number of effective alleles per locus.

^**c**^expected heterozygosity.

^**d**^Shannon’s information index.

^**e**^polymorphism information content.

A total of 285 alleles were detected in 40 diploid cultivars, the number of alleles per locus (*Ae*) ranged from 2 to 5, with a mean of 2.899. The expected heterozygosity (*He*) in the present study ranged from 0.049 to 0.788, with an average of 0.487 and Shannon’s information index (*I*) ranged from 0.117 to 1.578, with an average of 0.819 in diploid rice. Polymorphism information content (PIC) values were ranged from 0.048 to 0.753, with an average of 0.421.

In autotetraploid rice, 291 alleles were detected and the number of alleles per locus ranged from 2 to 5, with a mean frequency of 2.949. The expected heterozygosity ranged from 0.049 to 0.792, with an average of 0.493 and *I* was ranged from 0.117 to 1.664, with an average of 0.822, and PIC were ranged from 0.043 to 0.787, with an average of 0.432. Autotetraploid lines showed a higher number of *Ae*, *He*, *I* and PIC than diploid cultivars.

In addition, we found that 10 SSR markers showed more variation in the number of alleles among 99 SSR markers. In comparison with the diploid rice, seven markers RM443, RM22, RM559, PSM383, RM13, RM340 and PSM410 showed high number of alleles in autotetraploid rice, while RM282, RM257, RM258 showed more alleles in diploid rice. These results showed that there might be a genetic variation at DNA level, which leads to differentiation in the diploid and autotetraploid rice.

### Phylogenetic analysis of diploid and autotetraploid rice

**Figure 1:**
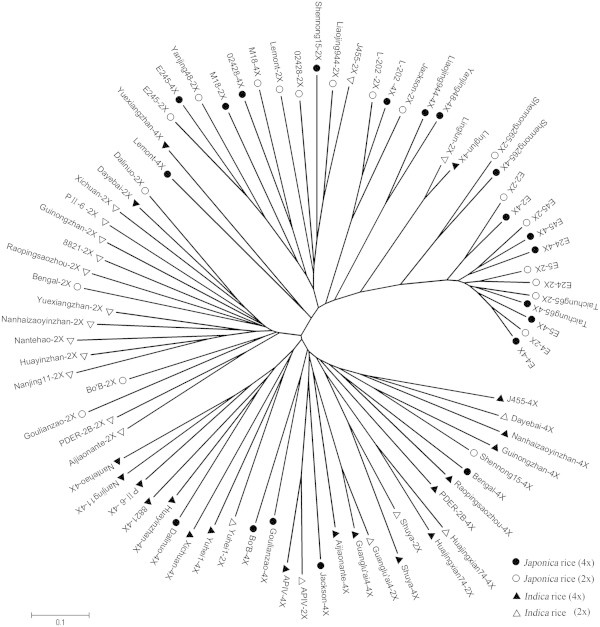
**Unrooted neighbor-joining tree based on SSR markers showing relationships among 80 autotetraploid and diploid rice cultivars.**

We constructed a phylogenetic tree based on the SSR markers to evaluate the genetic variation between diploid and autotetraploid rice because SSR markers have a higher resolution in separating the different rice cultivars. Thirteen pairs of diploid and autotetraploid rice showed high similarity and grouped into the same clade, while other 27 pairs of autotetraploid and corresponding diploid rice cultivars were phylogenetically distinct from other cultivars and clustered together on a distinct branch of phylogenetic tree (Figure 1).

All autotetraploid and diploid rice cultivars were distinguished very well and clustered into two main groups, which were subdivided into smaller groups. Group I consisted of 34 autotetraploid and diploid rice cultivars, mainly belong to *japonica* subspecies. We can clearly distinguished two subgroups in Group I: Subgroup I-A mainly included 32 autotetraploid and corresponding diploid rice cultivars. Taichung 65-2×/Taichung 65-4×, E2-2×/E2-4×, E4-2×/E4-4×, E45-2×/E45-4×, E245-2×/E245-4×, Shennong 265-2×/Shennong 265-4×, L-202-2×/L-202-4× and Linglun-2×/Linglun-4× showed closest relationship between diploid and autotetraploid rice, while E5-2×/E5-4×, E24-2×/E24-4×, Liaojing 944-2×/Liaojing 944-4×, Yanjing 48-2×/Yanjing 48-4× had most distant relationship and showed more variation in autotetraploid and diploid rice cultivars. Subgroup I-B only had 2 autotetraploid rice lines, which were Yuexiangzhan-4× and Lemont-4×, both of them had a distant relationship from their original diploid rice.

Group II consisted of 46 cultivars of diploid and autotetraploid rice and most of them belong to *indica* subspecies. This group could be further subdivided into three subgroups: Subgroup II-A consisted of 20 cultivars, Subgroup II-B consisted of 9 cultivars and Subgroup II-C consisted of 17 cultivars. In the group II, only 5 pairs of corresponding diploid and autotetraploid rice such as Huajingxian 74-2×/Huajingxian 74-4×, Shuya-2×/Shuya-4×, Guanglu’ai 4-2×/Guanglu’ai 4-4×, APIV-2×/APIV-4× and Yuhei 1-2×/Yuhei 1-4× showed the closest relationship with each other and other rice cultivars showed high variation and distant relationship between autotetraploid and their diploid counterparts.

### Heterosis analysis of diploid and autotetraploid rice

To evaluate the heterosis level of inter-subspecific autotetraploid hybrids, eleven parents were selected based on the genetic distance of *indica* and *japonica* autotetraploid rice. A total of fifty four *indica-japonica* hybrids were developed by crossing four typical *japonica* rice cultivars with seven *indica* rice cultivars, and their corresponding diploid parents were used as control (Table 2).

**Figure 2:**
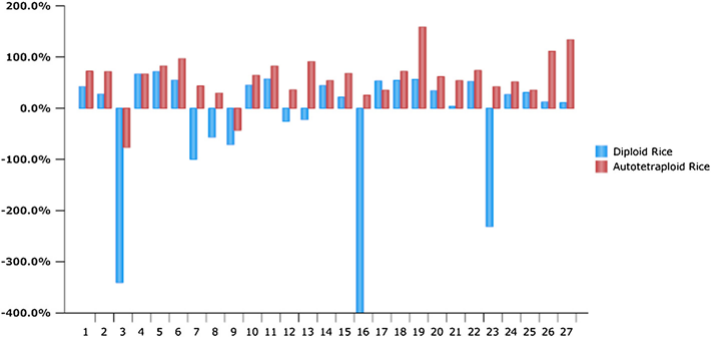
**Graphical representation of heterobeltiosis for grain yield in F**_**1**_**hybrids of autotetraploid and diploid rice.** Columns 1–27 indicate inter-subspecific hybrids of autotetraploid and their counterpart diploid genotypes: Liaojing 944 × Aijiaonante, Liaojing 944 × Guanglu’ai 4, Liaojing 944 × PDER-2B, Liaojing 944 × Raopingsaozhou, Liaojing 944 × Xichuan, Liaojing 944 × Dayebai, Yanjing 48 × Aijiaonante, Yanjing 48 × Guanglu’ai 4, Yanjing 48 × PDER-2B, Yanjing 48 × Raopingsaozhou, Yanjing 48 × Nanhaizaoyinzhan, Yanjing 48 × Xichuan, Yanjing 48 × Dayebai, Shennong 15 × Aijiaonante, Shennong 15 × Guanglu’ai 4, Shennong 15 × PDER-2B, Shennong 15 × Raopingsaozhou, Shennong 15 × Nanhaizaoyinzhan, Shennong 15 × Xichuan, Shennong 15 × Dayebai, Taichung 65 × Aijiaonante, Taichung 65 × Guanglu’ai 4, Taichung 65 × PDER-2B, Taichung 65 × Raopingsaozhou, Taichung 65 × Nanhaizaoyinzhan, Taichung 65 × Xichuan and Taichung 65 × Dayebai.

Positive heterobeltiosis analysis revealed that most autotetraploid hybrids showed superior results than corresponding better parents for most of the agronomic traits and grain yield (Table 5). Twenty-five F_1_ combinations showed significant and positive heterosis over the better parents in autotetraploid rice, and Shennong 15 × Xichuan was identified as the best specific combination with the highest level of heterobeltiosis in autotetraploid hybrids (Figure 2). GY showed the highest heterosis among all the traits, followed by GPP, PH, PL, SS, GWT, GD, EPN, GW, GL and L/W in autotetraploid rice. However the heterobeltiosis levels varied considerably in diploid rice, Liaojing 944 × Xichuan exhibited the highest heterobeltiosis among diploid rice curtivars. EPN produced the highest heterosis among the all traits observed, followed by GPP, GY, GWT, GD, PH, PL, GW, GL, L/W and SS in diploid rice.

**Table 5:** **Analysis of heterobeltiosis and competitive heterosis for important agronomic traits in autotetraploid and diploid rice**

Traits	Heterobeltiosis (compared with better parent)								Competitive heterosis			
	Diploid				Autotetraploid				(compared with corresponding diploid hybrids)			
	Minimum	Maximum	Mean	+ (−)^a^	Minimum	Maximum	Mean	+ (−)	Minimum	Maximum	Mean	+ (−)
	-------%-------				-------%-------				-------%-------			
PH	−13.56	25.74	3.52	18 (9)	−17.94	60.36	15.26	23 (4)	−20.77	27.43	−0.38	14 (13)
EPN	−42.00	147.83	13.83	14 (13)	−82.84	11.76	10.17	11(16)	−79.45	11.22	−35.78	1 (26)
PL	−21.65	20.07	−0.96	12 (15)	−13.06	40.17	13.01	24 (3)	3.24	38.99	17.60	27 (0)
GPP	−22.36	142.45	34.03	20 (7)	−63.39	50.29	17.91	18(9)	−80.73	−5.57	−46.30	0 (27)
GD	−41.46	30.01	0.22	11 (16)	−49.44	13.74	−26.31	1 (26)	−62.92	−13.88	−40.83	0 (27)
GL	−25.92	3.39	−5.08	3 (24)	−16.96	7.10	2.77	9 (18)	10.64	57.23	24.43	27 (0)
GW	−26.90	4.44	−3.86	8 (19)	−10.63	9.89	0.64	14 (13)	−0.91	40.67	13.88	26 (1)
L/W	−42.41	−2.51	−14.98	0 (27)	−33.63	−3.46	−18.42	0 (27)	−3.55	39.73	10.51	25 (2)
GWT	−4.98	33.32	10.96	16 (11)	−25.28	34.53	11.22	22 (5)	−10.52	55.32	30.80	27 (0)
GY	−663.68	71.90	−28.95	18 (9)	−76.82	158.78	71.16	25(2)	−27.45	89.85	21.14	26(1)
SS	−676.40	−19.31	−160.43	0 (27)	−332.66	36.14	−22.33	17 (10)	−289.71	78.64	2.01	19(8)

^a^ indicates increased (+) or decreased (−) effects in 27 hybrids.

*PH* = plant height, *PL* = panicle length, *EPN* = effective panicles number, *GL* = grain length, *GW* = grain width, *L/W* = grain length to width ratio, *GD* = grain density, *GPP* = grains per panicle, *GWT* = 1000-grain weight, *GY* = grain yield and SS = seed set ratio.

Competitive heterosis was used to further study the heterosis of autotetraploid and corresponding diploid rice hybrids (Table 5). For the competitive heterosis, most of agronomic traits such as PL (ranged from 3.24 to 38.99 with a mean of 17.60%), GL (ranged from 10.64 to 57.23 and 24.43%), GW (ranged from −0.91 to 40.67 with a mean of 13.88%), L/W (ranged from −3.55 to 39.73 with a mean of 10.51%), SS (ranged from −289.71 to 78.64 with a mean of 2.01%) and GWT (ranged from −10.52 to 55.32 with a mean of 30.8%) showed positive competitive heterosis or increased effects in autotetraploid rice hybrids among the agronomic traits. Yield also exhibited positive competitive heterosis and ranged from −27.45 to 89.85, with an average of 21.14%. However, PH (ranged from −20.77 to 27.43 with a mean of −0.38%), EPN (ranged from −79.45 to 11.22 with a mean of −35.78%), GPP (ranged from −80.73 to −5.57% with a mean of −46.30%) and GD (ranged from −62.92 to −13.88 with a mean of −40.83%) showed reduction in competitive heterosis in autotetraploid rice hybrids. Twenty two hybrids showed significant and positive heterosis over the diploid hybrids, whereas only five autotetraploid hybrids depicted reduction in competitive heterosis.

### Correlation analysis of yield and genetic diversity with agronomic traits

**Figure 3:**
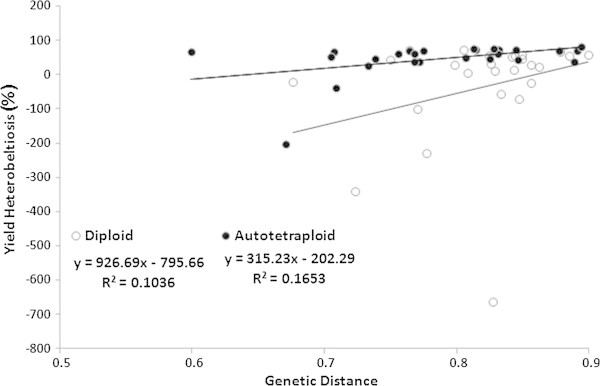
**A regression correlation analysis between genetic distance and yield heterobeltiosis in inter-subspecific hybrids of diploid and autotetraploid rice.**

Grain yield and genetic distances based on SSR markers were used for correlation with heterosis in diploid and autotetraploid rice (Table 6). The correlation between SSR marker distance and heterobeltiosis for all the agronomic traits was non-significant in diploid rice, indicating that prediction of hybrid performance using SSR markers in diploid rice is low. The relationship between genetic distance and heterobeltiosis for most of agronomic traits was also non-significant in autotetraploid rice. However, it was significantly and positively correlated for grain length (P < 0.01, 0.514) and grain length to width ratio (P < 0.05, 0.412) in autotetraploid hybrids. The linear regression analysis between genetic distance and yield heterobeltiosis was positive and non-significant with *R*^*2*^ value of 0.1036 in diploid rice, while significant and positive relationship was found in autotetraploid rice (Figure 3, Table 6). The correlations of grain yield heterobeltiosis with agronomic traits markedly differed in diploid and autotetraploid rice. For yield, autotetraploid rice showed significant correlations with EPN, GW and GWT, while yield showed significant correlations with GD and L/W in diploid rice.

## Discussion

### Genetic variation in diploid and autotetraploid rice

The analysis of genetic variation among different genotypes provides basic information about the germplasm enhancement for breeding. Autotetraploid rice with doubling of chromosomes showed a higher variation in agronomic traits, cellular level, quality and molecular level (Song and Zhang 1992;Luan et al. 2008). In the present study, autotetraploid rice had higher 1000-grain weight, grain length and grain width, but lower effective spikelet number, number of total panicles per plant and seed set ratio. In comparison with the diploid rice, production-related agronomic traits mainly occurred in autotetraploid rice. Therefore, 14 production-related agronomic traits were used to evaluate the phenotypic variation, and the results showed that there was a significant difference in diploid and autotetraploid rice for all agronomic traits under study. These results are in agreement with other studies, that autotetraploid showed significant variation in agronomic traits than their diploid counter parts (Tu et al. 2007;Shahid et al. 2011).

SSR markers showed a high polymorphism in the rice genome, which can offer unique opportunity for studying rice genotypes, genetic variation and genetic relationship (McCouch et al. 2002;Shah et al. 2013). Therefore, we used the SSR markers to evaluate the genetic variation of diploid and autotetraploid rice. The SSR results indicated that alleles per locus, expected heterozygosity, Shannon’s information index and polymorphism information contents were higher in autotetraploid than diploid rice. Moreover, phylogenetic tree was constructed based on SSR markers and it revealed that most of autotetraploid rice genotypes are genetically isolated from corresponding diploid cultivars, and only thirteen pairs of corresponding diploid and autotetraploid rice showed a high similarity phylogenetic relationship and grouped into the same clade. This finding is consistent with previous study that autotetraploid rice showed more genetic variation than diploid rice (Luan et al. 2008). Interestingly, both phenotypic and genotypic data showed a greater genetic variation in autotetraploid rice than diploid counterpart. These results suggested that there might be chromosome alteration or DNA sequence changes in autotetraploid rice that need further study, using functional molecular markers and SNP markers to analyze specific traits of autotetraploid rice.

### Heterosis and genetic relationship in autotetraploid rice

Hybrid breeding is one of the best techniques to increase the crop yield and it is successfully being used for many crops in China. Asian cultivated rice, *indica* and *japonica*, showed high hybrid vigor and numerous studies have been done to utilize the heterosis of these subspecies in diploid rice. However, little is known about autotetraploid rice and one of the major aim is the development of F_1_ hybrids to utilize the advantage of polyploidy and *indica-japonica* heterosis. Inter-subspecific autotetraploid rice hybrids had shown stronger yield potential and greater adaptability compared with diploid rice (Shahid et al. 2011). Previous studies demonstrated high heterosis for panicles, 1000-grain weight, grain length and grain width in autotetraploid rice (Shahid et al. 2011,2012) and three-line hybrid system had been already established in autotetraploid rice (Tu et al. 2007). Therefore, we selected several typical *indica* and *japonica* autotetraploid and diploid rice cultivars based on genetic distance of *indica* and *japonica* rice varieties to study the heterosis in autotetraploid rice. Higher competitive heterosis and heterobeltiosis was found in autotetraploid than diploid rice. Heterobeltiosis for most of the agronomic traits, including PH, PL, GL, SS, GW, GY and GWT, was positive and showed a higher proportion in autotetraploid hybrids than diploid hybrids. In general, the results of the present study were in agreement with earlier investigations for autotetraploid rice hybrids (Tu et al. 2007;Shahid et al. 2011). In addition, autotetraploid rice hybrids also showed positive competitive heterosis or increased effects among the agronomic traits than diploid rice hybrids. For example, all autotetraploid hybrids (except one) showed significant and positive heterosis over the corresponding diploid hybrids for grain yield. From these results, we speculated that higher genetic variation in autotetraploid rice might be the result of changes in DNA structure of autotetraploid rice.

**Table 6:** **Correlation coefficients of grain yield and genetic distance with important agronomic traits on the basis of heterobeltiosis in diploid and autotetraploid rice**

Traits	Grain Yield		Genetic distance	
	Diploid	Autotetraploid	Diploid	Autotetraploid
**PH**	0.040	−0.051	−0.014	0.246
**EPN**	−0.151	0.541**	−0.253	−0.072
**PL**	0.005	0.114	−0.180	0.221
**TGP**	0.178	0.152	−0.256	0.102
**GD**	0.586**	0.186	0.292	−0.191
**GL**	−0.097	−0.057	0.064	0.514**
**GW**	−0.198	0.367*	−0.112	−0.181
**L/W**	−0.389*	−0.263	−0.009	0.412*
**GWT**	0.349	0.528**	0.088	0.076

*,** Significantly different from zero at P < 0.05 and P < 0.01, respectively.

See Table 5 for traits abbreviations.

To increase the hybrid breeding efficiency, DNA markers have been used to investigate the parental genetic distance and its relationship with heterosis (Caruso et al. 2010). Previous studies showed that heterosis mainly depends on the genetic variation and traits of the parent’s interaction, the greater the genetic variation and genetic distance, the more obvious hybrid vigour (Zhang et al. 1994). Numerous studies are available for evaluating usefulness of DNA markers for predicting heterosis and hybrid performance in diploid rice and other crops. There are two contradicting theories about heterosis prediction, some scientists suggested that molecular markers could be used for predicting heterosis (Smith et al. 1990;Zhang et al. 1994;Zha et al. 2008;Jaikishan et al. 2010), while other suggested that heterosis could not be predicted through molecular markers (Joshi et al. 2001). In the present study, SSR markers were employed to predict the heterosis in autotetraploid and diploid rice. The results from this study demonstrated non-significant correlation between genetic distance and heterosis for all agronomic traits and yield in diploid rice. This finding is consistent with other conclusions on the relationship between genetic distance and heterosis; especially in inter-subspecific hybrids of rice (Xiao et al. 1996;Zhang et al. 2007;Xangsayasane et al. 2010). SSR markers-based genetic distance might not be a reliable tool in hybrid breeding. Non-significant relationship between SSR markers diversity and heterosis could be because SSR diversity represented a genome-wide diversity, whereas heterozygous loci for each trait could be localized to a specific region (Jaikishan et al. 2010). In this study, correlation between molecular marker distance and yield heterobeltiosis was significant in autotetraploid rice. Grain length and grain length to width ratio also depicted significant relationship with genetic distance, while all other traits showed non-significant relationships. These results are in agreement with previous reports on some other polyploid crops such as wheat, cotton, sugarcane and Indian mustard, who also found significant correlation between markers diversity and some traits understudy (Martin et al. 1995;Zhang et al. 2007). The maximum heterobeltiosis was recorded from genetically far distant autotetraploid and diploid parents. There had been no investigations to assess the relationship between genetic distance and hybrid performance in autotetraploid rice. In the present study, molecular markers were not suitable for prediction of hybrid performance for most of the traits, however, marker-based genetic distance showed a significant relation with yield heterosis, grain length and grain length to width ratio in autotetraploid parents. The information generated from this study will be useful for future autotetraploid rice breeding plans.

In summary, SSR markers are very useful to find genetic variation and phylogenetic analysis in different ploidy level, but they are not a reliable tool to predict heterosis for yield and other complex traits in diploid rice. Autotetraploid lines showed greater genetic differentiations which we can’t find in diploid rice and both have a marked difference in their gene pool. Therefore, these results suggest that autotetraploid rice is an important germplasm for breeding and molecular studies. We could improve the rice cultivars through autotetraploid rice breeding for various important traits because autotetraploid rice had great stability across varying environments, resistant to lodging, greater grain length and width and resistant to insect pest and diseases. This may be an advantage to breed higher yield and better quality rice through autotetraploid rice breeding.

## Abbreviations

*Ae*: Number of effective alleles per locus
EPN: Effective panicles number
FLL: Flag leave length
FLW: Flag leave width
GD: Grain density
GL: Grain length
GPP: Grains per panicle
GW: Grain width
GWT: 1000-grain weight
GY: Grain yield
He: Expected heterozygosity
L/W: Grain length to width ratio
PH: Plant height
PL: Panicle length
PIC: Polymorphism information content
TGP: Total number of grains per plant
I: Shannon’s information index
SS: Seed set ratio.
